# Lorlatinib in advanced ALK-positive NSCLC after prior progression on ALK inhibitors: real-world experience in Russia

**DOI:** 10.37349/etat.2026.1002366

**Published:** 2026-04-16

**Authors:** Sergey V. Orlov, Konstantin K. Laktionov, Aram A. Musaelyan, Elena V. Reutova, Svetlana V. Odintsova, Magaripa A. Urtenova, Valeria A. Kuzmina, Vladislav I. Tiurin, Alexandra E. Lomakova, Evgeny N. Imyanitov

**Affiliations:** Università degli Studi della Campania “Luigi Vanvitelli”, Italy; ^1^Department of Clinical Oncology, Pavlov First Saint Petersburg State Medical University, St. Petersburg 197022, Russia; ^2^EuroCityClinic LLC, St. Petersburg 197022, Russia; ^3^I.V. Kurchatov Complex for Medical Primatology, National Research Centre “Kurchatov Institute”, Sochi 354376, Russia; ^4^N.N. Blokhin National Medical Research Center of Oncology, Ministry of Health of the Russian Federation, Moscow 115522, Russia; ^5^Department of Tumor Growth Biology, N.N. Petrov National Medical Research Center of Oncology, Ministry of Health of the Russian Federation, St. Petersburg 197758, Russia; ^6^Department of Medical Genetics, St.-Petersburg State Pediatric Medical University, St. Petersburg 194100, Russia

**Keywords:** real-world evidence, lorlatinib, ALK, tyrosine kinase inhibitor, NSCLC, efficacy, safety

## Abstract

**Aim:**

This study aimed to evaluate the real-world efficacy and safety of lorlatinib in patients with anaplastic lymphoma kinase (ALK)-rearranged metastatic non-small cell lung cancer (NSCLC) after the failure of at least one prior ALK tyrosine kinase inhibitor (TKI).

**Methods:**

The dataset included 82 subjects with metastatic NSCLC, who received lorlatinib upon compassionate use program or routine treatment between January 2017 and May 2025. All patients involved in this study responded to a prior ALK inhibitor for at least 4 months and switched to the above drug due to disease progression.

**Results:**

The overall objective response rate (ORR) was 64.6%, with the disease control rate (DCR) of 96.3%. Among 65 patients with brain metastases, the intracranial ORR and DCR were 66.2% and 96.9%, respectively. After a median follow-up of 82.7 months, the median progression-free survival (PFS) was 66.7 months (95% CI, 40.5–75.0 months), while the median overall survival (OS) was not reached (NR) (95% CI, NR–NR). Patients who had benefited from prior ALK TKI for more than 12 months achieved significantly longer PFS (NR vs. 34.0 months; *p* = 0.013) and OS (NR vs. 39.4 months; *p* = 0.002). Multivariate analysis showed that prior response to ALK TKI of less than 12 months was an independent negative predictor of survival (PFS: *p* = 0.039, OS: *p* = 0.027). Treatment-related adverse events (AEs) were reported in 75.6% of patients, with 8.1% experiencing grade 3 or higher toxicity; no treatment-related AEs led to permanent discontinuation of lorlatinib.

**Conclusions:**

This real-world dataset demonstrates an unusually pronounced benefit from lorlatinib in selected patients who progressed on early-generation TKIs, especially in long-term responders to prior therapy. However, the observed outcomes should be interpreted within the context of patient selection. The enrichment for prior responders limits the generalizability to unselected post-TKI populations, including those with primary resistance.

## Introduction

Anaplastic lymphoma kinase (ALK) fusions account for approximately 4–5% of non-small cell lung cancer (NSCLC) cases and are particularly enriched among younger patients and never-smokers [1, 2]. These rearrangements lead to constitutive activation of ALK kinase signaling, which drives the NSCLC progression [1]. The first-generation ALK tyrosine kinase inhibitor (TKI), crizotinib, produces objective response in 65–74% of patients [3]; however, most patients develop acquired resistance within the first year of treatment [4]. Short progression-free survival (PFS) on crizotinib is attributed to the rapid emergence of secondary *ALK* mutations and insufficient penetration of the drug to the central nervous system (CNS) [3].

Second-generation ALK inhibitors, such as alectinib, ceritinib, brigatinib, and ensartinib, were developed to address these limitations [2]. These agents have shown significantly improved survival outcomes over crizotinib, increased activity toward CNS metastases, and the ability to overcome some crizotinib-resistant mutations [5]. However, resistance eventually develops even with second-generation ALK TKIs, highlighting the need for further advancements in ALK-targeted therapy [5, 6].

Lorlatinib is a third-generation, macrocyclic TKI that targets ALK and ROS1 [7]. It has a high potential for penetrating the CNS and effectively counteracting most single-point *ALK* resistance mutations, such as *G1202R* [5, 7]. A phase II study enrolled patients with ALK-positive NSCLC who had progressed on one or more prior ALK inhibitors [7]. In this heavily pretreated population, lorlatinib achieved an objective response rate (ORR) of 47%, with particularly striking activity in patients with CNS metastases, where intracranial response rates reached 63% [7]. In the CROWN phase III trial, lorlatinib showed unprecedented efficacy when administered as an upfront treatment [8]. After 5 years of follow-up, the lorlatinib arm still did not reach the median PFS, which is apparently the best achievement in the history of systemic therapy of common solid tumors [8].

Despite the advantage of lorlatinib over other ALK-targeted drugs, immediate global switch to this compound appears to be complicated due to several practical reasons. First, a substantial proportion of patients with ALK-rearranged NSCLC currently continue successful treatment by 1st- and 2nd-generation ALK TKIs, as all the drugs mentioned above produce substantial rates of very prolonged responses. Secondly, many countries have limited access to lorlatinib due to financial or regulatory obstacles. Third, an unusual spectrum of adverse effects of lorlatinib, including a risk of psychiatric disorders and a probability of weight gain, needs to be considered while projecting individual trajectories of treatment for NSCLC patients. Therefore, although lorlatinib may be considered as a preferred option in the first-line setting [9, 10], many NSCLC patients will remain on treatment with other ALK inhibitors in the next years; therefore, the accumulation of relevant real-world clinical evidence is warranted. In the present study, we retrospectively analyzed the efficacy and safety of lorlatinib in metastatic NSCLC patients who were treated with lorlatinib after prior progression on other ALK-targeted therapies.

## Materials and methods

The study included 82 patients with *ALK*-positive metastatic NSCLC, who received lorlatinib through an early access program or routine clinical practice between January 2017 and May 2025 at the Pavlov First Saint Petersburg State Medical University, the EuroCityClinic LLC (both in St. Petersburg, Russia), or the N.N. Blokhin National Medical Research Center of Oncology (Moscow, Russia). The study protocol was approved by the Ethics Committee of Pavlov First Saint Petersburg State Medical University (approval no. 05/25) and was conducted in accordance with the Declaration of Helsinki. All participants signed written informed consent forms. All patients were initially treated with at least one line of first-generation (crizotinib) and/or second-generation ALK TKI (alectinib, ceritinib, brigatinib), and later experienced disease progression on the therapy. These subjects received lorlatinib at a daily dose of 100 mg until progression or unacceptable toxicity.

**Figure 1:**
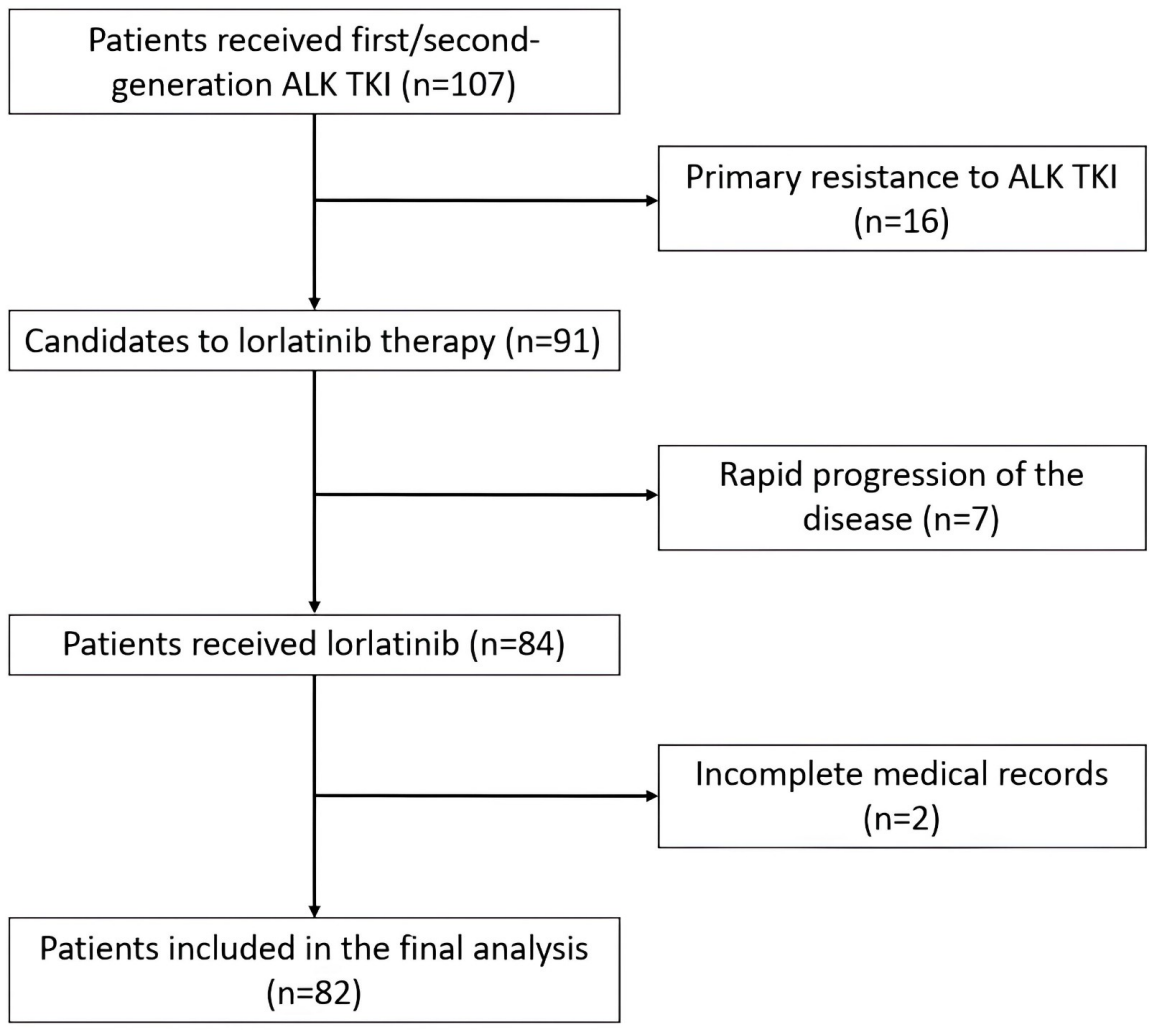
**Flow diagram of patients included in the analysis of the study.** ALK: anaplastic lymphoma kinase; TKI: tyrosine kinase inhibitor.

The inclusion criteria were as follows: (1) patients aged ≥ 18 years with histologically confirmed metastatic NSCLC harboring an *ALK* rearrangement, as determined by polymerase chain reaction (PCR), next-generation sequencing (NGS), fluorescence in situ hybridization (FISH), or immunohistochemistry (IHC); (2) Eastern Cooperative Oncology Group Performance Status (ECOG PS) scale of 0–2; (3) received at least one prior ALK TKI with a documented objective response (complete response, partial response or stable disease) lasting ≥ 4 months, followed by disease progression; (4) available comprehensive clinical data in medical records; (5) at least one post-baseline radiological assessment while receiving lorlatinib. The exclusion criteria were as follows: (1) no prior exposure to ALK TKIs; (2) primary resistance to prior ALK TKI (progression within 4 months of initiation without objective response or stable disease); (3) concomitant use of other investigational agents during lorlatinib therapy; (4) incomplete medical records. Figure 1 displays the flow diagram of patient selection for the study analyzing the efficacy and safety of lorlatinib.

The efficacy of the drug was assessed according to the Response Evaluation Criteria in Solid Tumors version 1.1 (RECIST 1.1). The intracranial response was also evaluated using RECIST 1.1 during magnetic resonance imaging at standardized intervals. The National Cancer Institute Common Terminology Criteria for Adverse Events version 5.0 (CTCAE v5.0) was used to grade adverse events (AEs).

Statistical analyses were conducted using GraphPad Prism 9.5.0 (GraphPad Software, Inc.). Categorical variables were compared using Fisher’s exact test. Survival outcomes were visualized with Kaplan-Meier curves and compared using the log-rank test. The median follow-up was evaluated using the reverse Kaplan-Meier method. Two-sided testing was conducted with a significance threshold of *p* < 0.05 for all comparative analyses.

## Results

### Patient data

Baseline characteristics of the patients are summarized in Table 1. It is essential to acknowledge that all patients from this dataset had a response to prior TKI for at least 4 months. The majority of patients [65/82 (79.3%)] had metastatic brain involvement at the beginning of lorlatinib treatment.

**Table 1:** The main baseline parameters of patients included in the study.

**Parameters**	**Study cohort (*n* = 82)**
Sex	*n* (%)
male	39 (47.6)
female	43 (52.4)
Age at initial diagnosis, median (range), years	47 (24–83)
Age at the start of lorlatinib, median (range), years	51 (25–85)
ECOG PS at lorlatinib initiation	*n* (%)
0/1	66 (80.5)
2	16 (19.5)
Smoking history	*n* (%)
Never smoker	54 (65.9)
Former smoker	23 (28.0)
Current smoker	5 (6.1)
Histology	*n* (%)
Adenocarcinoma	80 (97.6)
Non-adenocarcinoma NSCLC	2 (2.4)
Method of ALK identification	*n* (%)
PCR or NGS	36 (43.9)
FISH	37 (45.1)
IHC	9 (11.0)
Stage of disease at initial diagnosis	*n* (%)
I–II	5 (6.1)
III–IV	77 (93.9)
Brain metastases at lorlatinib initiation	*n* (%)
Yes	65 (79.3)
No	17 (20.7)
Number of brain metastases at lorlatinib initiation	*n* (% of total cases with brain metastases)
Single	11 (16.9)
Multiple	54 (83.1)
Local brain-directed therapy before the start of lorlatinib	*n* (% of total cases with brain metastases)
Stereotactic radiosurgery	22 (33.8)
Whole brain radiation therapy	14 (21.5)
None	29 (44.6)
Chemotherapy in prior lines	*n* (%)
Yes	57 (69.5)
No	25 (30.5)
Number of prior ALK TKIs	*n* (%)
1 (with crizotinib)	44 (53.7)
1 (with second-generation ALK TKI)	18 (22.0)
2	16 (19.5)
3	4 (4.9)
Last therapy before lorlatinib	*n* (%)
Crizotinib	44 (53.7)
Ceritinib	22 (26.8)
Brigatinib	11 (13.4)
Alectinib	5 (6.1)
Response to prior TKI	*n* (%)
≥ 4 and < 12 months	31 (37.8)
≥ 12 months	51 (62.2)
Line of lorlatinib therapy	*n* (%)
2	20 (24.4)
3	37 (45.1)
4	15 (18.3)
5	10 (12.2)

ALK: anaplastic lymphoma kinase; ECOG PS: Eastern Cooperative Oncology Group Performance Status; FISH: fluorescence in situ hybridization; IHC: immunohistochemistry; NGS: next-generation sequencing; NSCLC: non-small cell lung cancer; PCR: polymerase chain reaction; TKI: tyrosine kinase inhibitor.

### Response rates and disease control rates on lorlatinib therapy

The overall ORR for patients who received lorlatinib was 53/82 (64.6%), and the disease control rate (DCR) was 79/82 (96.3%) (Table 2). Among these cases, 5 patients had a complete response, 48 patients had a partial response, and 26 patients had stable disease. The ORR for patients with visceral only metastases and for patients with CNS metastases was 8/17 (47.1%) and 43/65 (66.2%), respectively (*p* = 0.169). The ORR for patients who received one prior ALK TKI was 62.9% (39/62), while patients who received two or more lines of ALK TKIs had an ORR of 70.0% (14/20) (*p* = 0.604). The ORR for patients who received crizotinib prior to lorlatinib was 28/44 (63.6%); this estimate was 15/22 (68.2%) for patients who progressed on ceritinib, 7/11 (63.6%) for subjects with prior brigatinib, and 3/5 (60%) for patients who failed on alectinib (*p* = 0.979). Patients who had responded to prior ALK TKI therapy for more than 12 months had an ORR of 35/51 (68.6%), while the ORR for individuals who had responded to prior targeted therapy for 4–12 months or less was 18/31 (58.1%) (*p* = 0.351).

**Table 2:** Evaluation of the tumor’s response to lorlatinib.

**Efficacy endpoints**	**Overall**	**Number of prior ALK TKI**			**Response to prior ALK TKI**		
		**1 line**	**≥ 2 lines**	***p***	**≤ 12 months**	**> 12 months**	***p***
ORR, *n* (%)	53/82 (64.6)	39/62 (62.9)	14/20 (70.0)	0.604	18/31 (58.1)	35/51 (68.6)	0.351
DCR, *n* (%)	79/82 (96.3)	60/62 (96.8)	19/20 (95.0)	1.000	28/31 (90.3)	51/51 (100.0)	0.051
IC-ORR, *n* (%)	43/65 (66.2)	34/49 (69.4)	9/16 (56.3)	0.372	14/25 (56.0)	29/40 (72.5)	0.190
IC-DCR, *n* (%)	63/65 (96.9)	47/49 (95.9)	16/16 (100.0)	1.000	23/25 (92.0)	40/40 (100.0)	0.144

ALK: anaplastic lymphoma kinase; ORR: objective response rate; DCR: disease control rate; IC-ORR: intracranial objective response rate; IC-DCR: intracranial disease control rate; TKI: tyrosine kinase inhibitor.

No statistically significant association was observed between ORR/DCR and various clinical characteristics, such as sex, age, ECOG PS, smoking status, stage at initial diagnosis, method of *ALK* detection, chemotherapy in prior lines, number of previous lines of ALK TKI, previously CNS-directed radiotherapy, last therapy before lorlatinib, and the line of lorlatinib therapy (*p* > 0.05).

The intracranial ORR (IC-ORR) for patients who had brain metastases at the start of lorlatinib therapy was 43/65 (66.2%) (14 patients with complete response and 29 subjects with partial response). The IC-ORR for patients who previously received one line of ALK TKIs was 34/49 (69.4%), while for individuals who had experienced progression on at least two ALK TKIs, it was 56.3% (9/16) (*p* = 0.372). The IC-ORR for patients who had previously received therapy with crizotinib, ceritinib, and brigatinib was 22/44 (50.0%), 16/22 (72.7%), and 5/11 (45.5%), respectively. Notably, none of the five patients with prior alectinib demonstrated intracranial tumor response (0/5 vs. 43/60, *p* = 0.155). Patients who had previously received ALK TKI therapy for more than 12 months had the IC-ORR 14/25 (56.0%), while the IC-ORR for those who had responded to prior targeted therapy for 12 months or less was 29/40 (72.5%) (*p* = 0.189). The IC-ORR for patients who previously received local CNS-directed therapy was 19/36 (52.8%), while for those who had not been given radiotherapy, it was 17/29 (58.6%) (*p* = 0.802).

The intracranial DCR (IC-DCR) in patients with brain lesions who received lorlatinib was 63/65 (96.9%). No statistically significant associations were observed between intracranial response outcomes (IC-ORR or IC-DCR) and clinical variables such as sex, age, ECOG PS, smoking history, stage at diagnosis, prior chemotherapy, prior CNS-directed radiotherapy, number of prior ALK TKIs, the most recent ALK TKI before lorlatinib, or line of lorlatinib therapy (*p* > 0.05).

### Survival outcomes with lorlatinib

**Figure 2:**
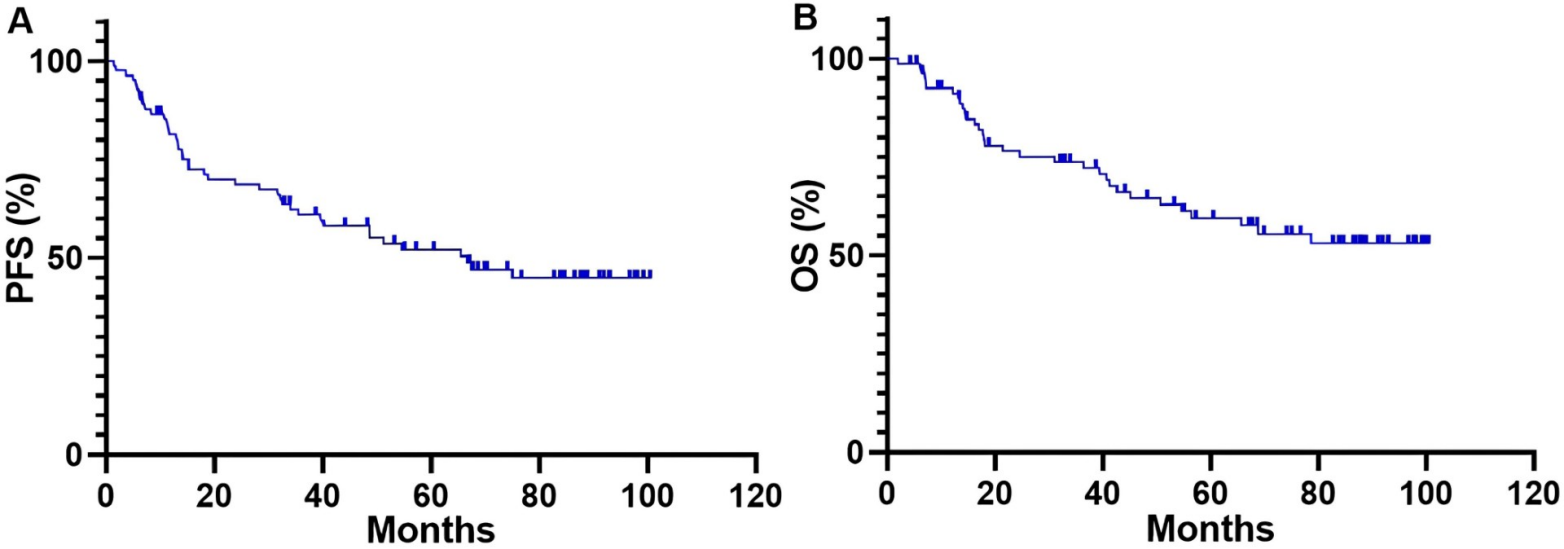
**Kaplan-Meier curves for PFS (A) and OS (B) of patients who received lorlatinib therapy.** PFS: progression-free survival; OS: overall survival.

The median follow-up for patients with *ALK*-positive metastatic NSCLC who were treated with lorlatinib was 82.7 months (95% CI, 67.7–87.8 months). At the data cutoff in May 2025, events (progression or death) occurred in 50% (41/82) of patients, while the remaining 50% (41/82) of patients were still on lorlatinib without progression. For overall survival (OS) analysis, deaths were observed in 39.0% (32/82) of cases. The median duration of lorlatinib exposure was 66.7 months (range 1.5–100.4). The median PFS for lorlatinib therapy was 66.7 months (95% CI, 40.5–75.0 months; Figure 2A), and the median OS was not reached (NR) (95% CI, NR–NR; Figure 2B).

**Figure 3:**
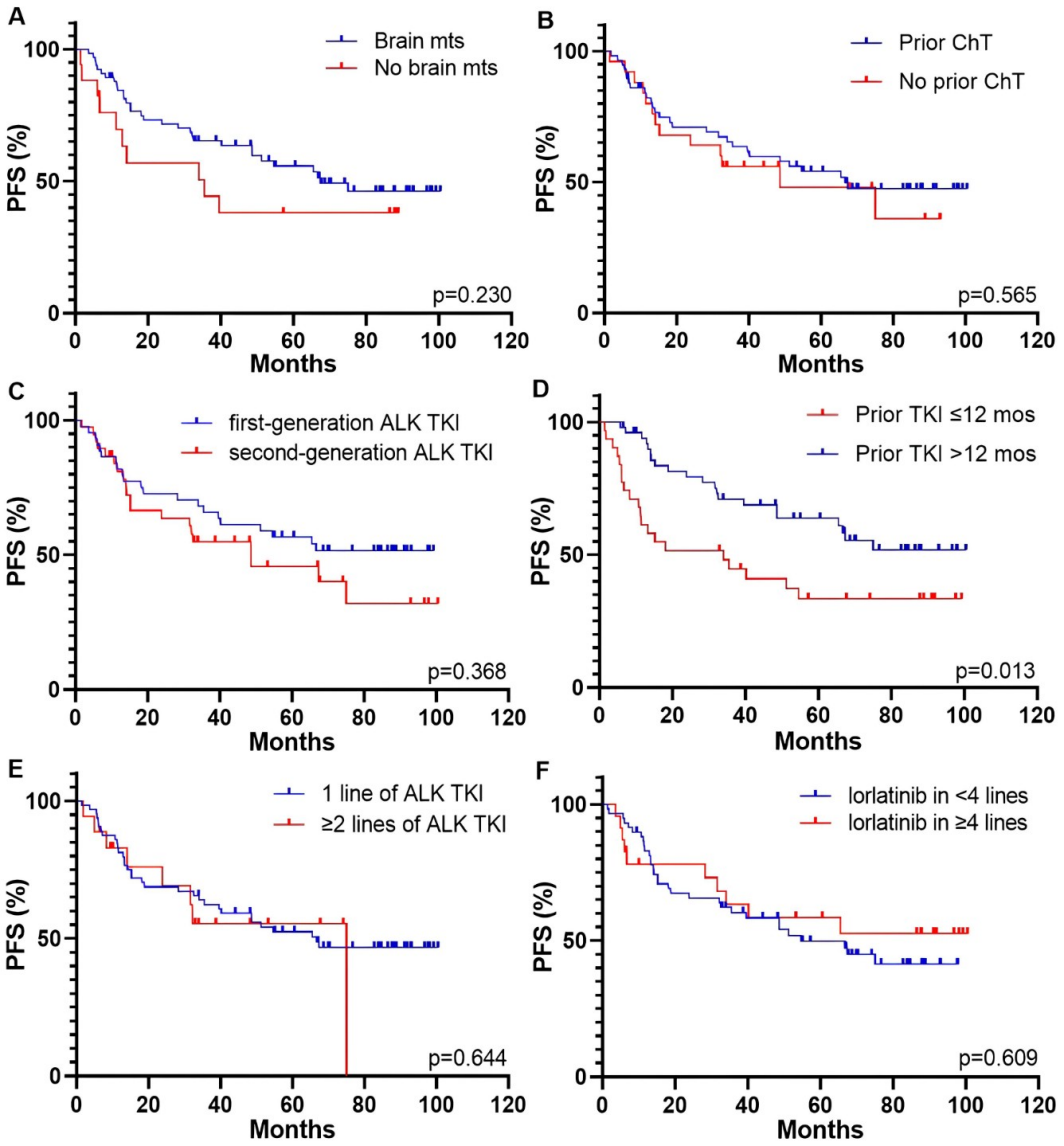
**Kaplan-Meier curves for PFS of patients who received lorlatinib therapy based on different parameters.** (**A**) brain mts; (**B**) prior chemotherapy; (**C**) generation of previous ALK TKI; (**D**) response to prior ALK TKI; (**E**) number of prior lines of ALK TKIs; (**F**) line of lorlatinib therapy. ALK: anaplastic lymphoma kinase; ChT: chemotherapy; mos: months; mts: metastases; PFS: progression-free survival; TKI: tyrosine kinase inhibitor.

**Figure 4:**
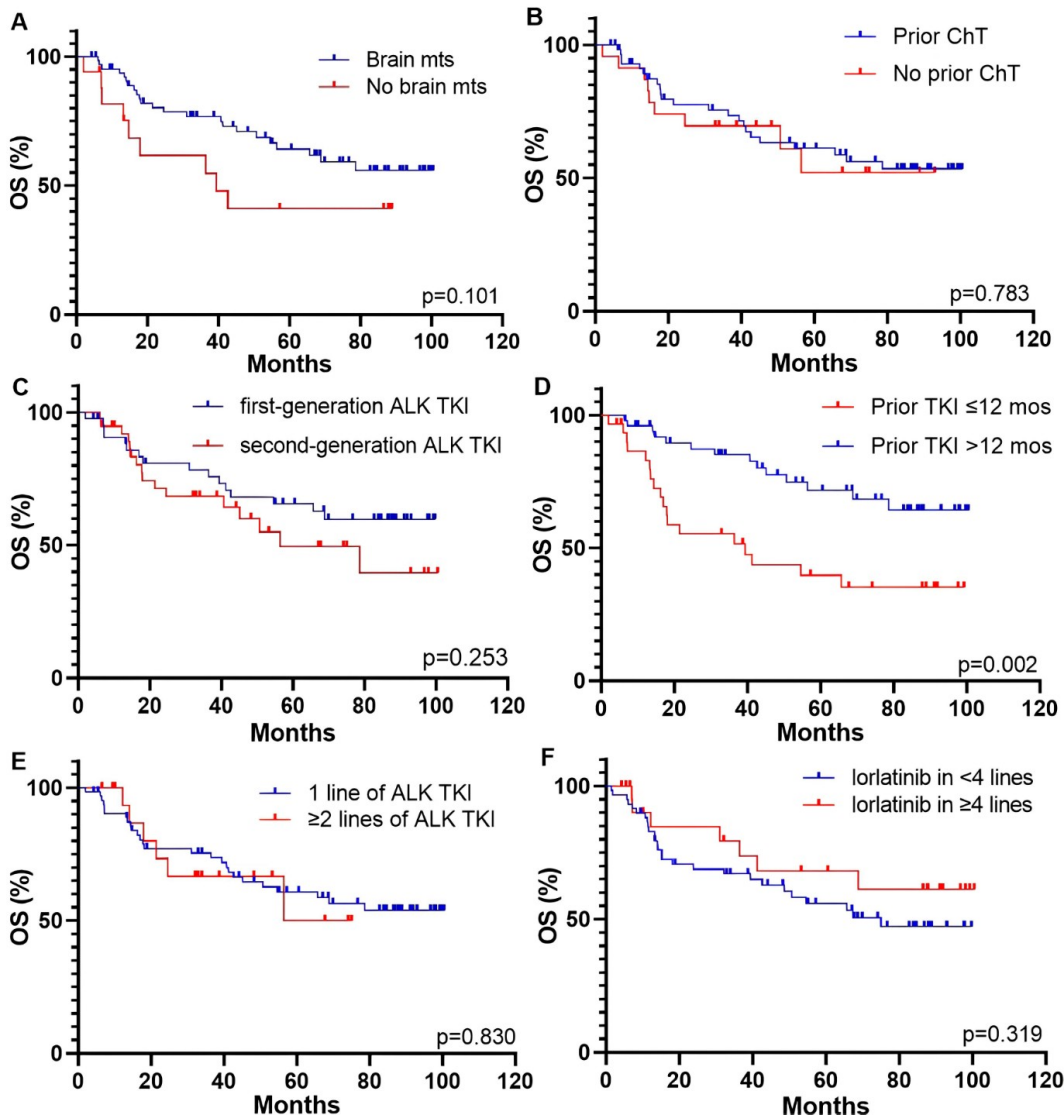
**Kaplan-Meier curves for OS of patients received lorlatinib therapy based on different parameters.** (**A**) brain mts; (**B**) prior chemotherapy; (**C**) generation of previous ALK TKI; (**D**) response to prior ALK TKI; (**E**) number of prior lines of ALK TKIs; (**F**) line of lorlatinib therapy. ALK: anaplastic lymphoma kinase; ChT: chemotherapy; mos: months; mts: metastases; OS: overall survival; TKI: tyrosine kinase inhibitor.

Patients with CNS metastases at the start of lorlatinib therapy had longer PFS and OS compared to those without brain involvement, although the difference was not statistically significant [PFS: 67.4 (95% CI, 48.7–75.0 months) vs. 35.5 months (95% CI, 6.7–39.5 months), *p* = 0.230, Figure 3A; OS: NR (95% CI, NR–NR) vs. 39.4 months (95% CI, 13.3–42.6 months), *p* = 0.101, Figure 4A]. Previous systemic chemotherapy did not affect survival outcomes on lorlatinib treatment. The median PFS for patients who had received prior platinum-based doublet and for those who had not received chemotherapy was 66.7 (95% CI, 35.4–67.4 months) and 48.7 months (95% CI, 15.2–75.0 months), respectively (*p* = 0.565; Figure 3B). The median OS was NR in both groups (for both cohorts—95% CI, NR–NR, *p* = 0.783, Figure 4B).

Patients who had previously received only crizotinib but not other ALK inhibitors showed longer PFS and OS compared to those who had prior treatment with the second-generation drugs, although these differences were not statistically significant [PFS: NR (95% CI, NR–NR) vs. 48.7 months (95% CI, 15.2–75.0 months), *p* = 0.368, Figure 3C; OS: NR (95% CI, NR–NR) vs. 56.4 months (95% CI, 40.6–78.6 months), *p* = 0.253, Figure 4C]. Patients who had responded to prior ALK TKI therapy for period exceeding 12 months had significantly longer PFS and OS on lorlatinib compared to patients who failed on previous ALK TKI within 12 months or less [PFS: NR (95% CI, NR–NR) vs. 34.0 months (95% CI, 16.7–54.6 months), *p* = 0.013, Figure 3D; OS: NR (95% CI, NR–NR) vs. 39.4 months (95% CI, 25.2–65.7 months), *p* = 0.002, Figure 4D].

There were no differences in survival outcomes observed between patients who experienced progression on one line of ALK TKI and those who had previously received at least two lines of ALK inhibitors [PFS: 66.7 (95% CI, 35.4–67.4 months) vs. 75.0 months (95% CI, 37.5–80.0 months), *p* = 0.644, Figure 3E; OS: NR (95% CI, NR–NR) vs. 65.7 months (95% CI, 27.8–68.5 months), *p* = 0.830, Figure 4E]. The optimal cutoff value for the number of lines of systemic therapy was defined as 4 or more. Patients who received lorlatinib treatment in less than 4 lines showed no differences in survival data compared to those who received treatment with lorlatinib in 4 or more lines [PFS: 54.6 months (95% CI, 32.5–75.0 months) vs. NR (95% CI, NR–NR), *p* = 0.609, Figure 3F; OS: 75 months (95% CI, 42.6–75.0 months) vs. NR (95% CI, NR–NR), *p* = 0.319, Figure 4F].

**Figure 5:**
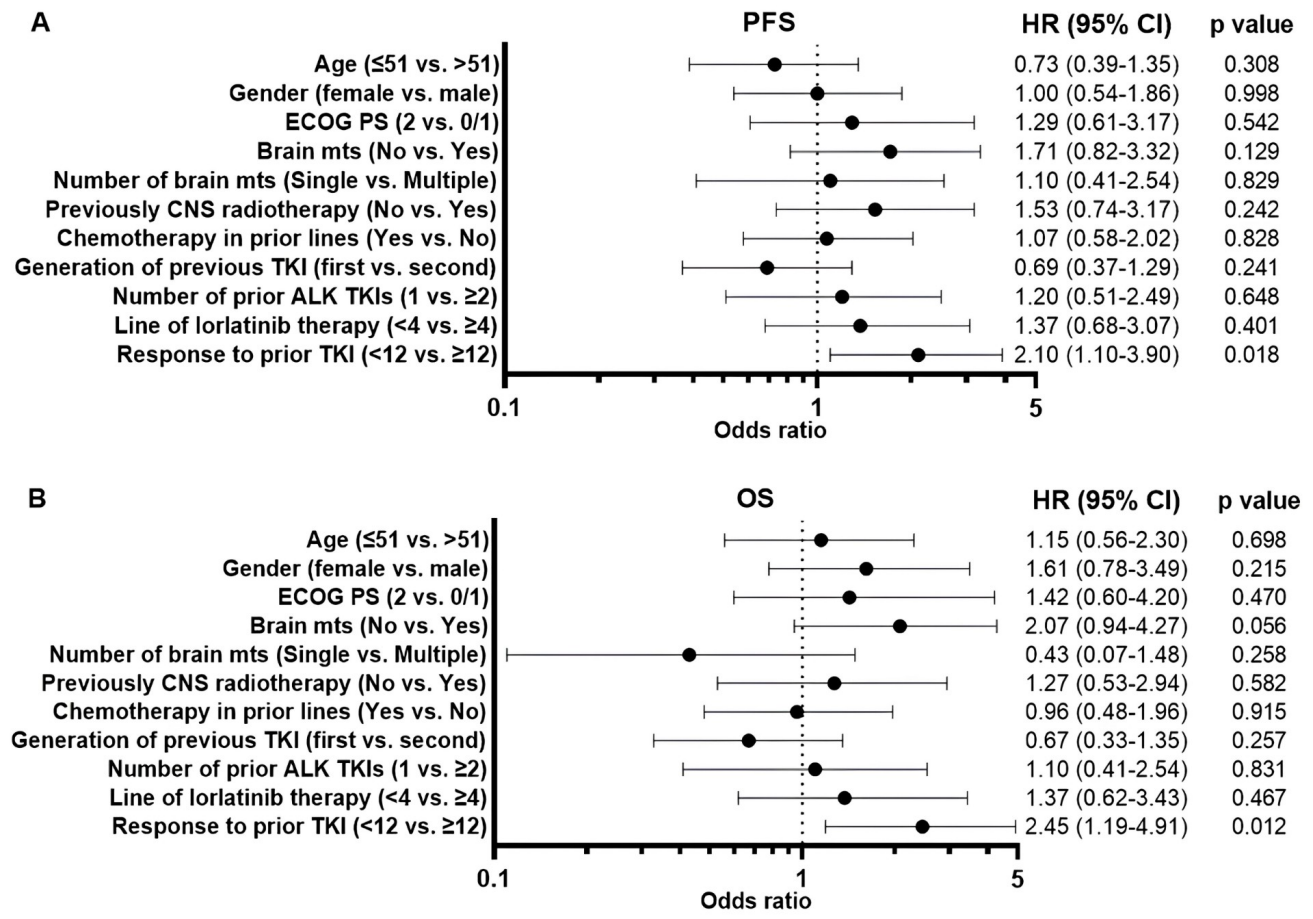
**The forest plots summarize the univariate analysis of clinical parameters for predicting progression-free survival (A) and overall survival (B).** ALK: anaplastic lymphoma kinase; CNS: central nervous system; ECOG PS: Eastern Cooperative Oncology Group Performance Status; mts: metastases; TKI: tyrosine kinase inhibitor.

In univariate analysis, prior ALK TKI benefit < 12 months was significantly associated with shorter PFS [hazard ratio (HR), 2.10; 95% CI, 1.10–3.90, *p* =0.018, Figure 5A] and OS (HR, 2.45; 95% CI, 1.19–4.91, *p* = 0.012, Figure 5B). The multivariate analysis revealed that response to prior ALK TKIs less than 12 months retained a statistically significant association with a negative survival outcome (PFS: HR, 1.97; 95% CI, 1.04–3.69, *p* = 0.039; OS: HR, 2.31; 95% CI, 1.08–4.85, *p* = 0.027).

### Safety profile of lorlatinib

Treatment-related AEs of any grade were observed in 75.6% (62/82) of patients who received lorlatinib. Grade ≥ 3 AEs were reported in 8.1% (5/62) of patients experiencing AEs. Dose reduction due to treatment-related AEs was reported in 17.1% (14/82) of patients. None of the patients permanently discontinued lorlatinib therapy due to toxicity.

The most common AEs were hyperlipidemia (56.1%; 46/82), peripheral edema (20.7%; 17/82), and neurocognitive disorders (8.5%; 7/82). Hyperlipidemia developed within 2–4 weeks of starting lorlatinib. Lipid-lowering therapy was started as per local protocols for grade ≥ 2 hyperlipidemia; the AE was reversible, with targeted therapy dose reduction in three cases. Peripheral edema occurred at a median of 2 months and was managed with diuretics, with two cases requiring dose reduction. Neurocognitive events developed within one month of lorlatinib initiation and were categorized as cognitive disturbance (43%, 3/7), mood event (43%, 3/7), and psychosis (14%, 1/7). All neurocognitive events were reversible and managed symptomatically, with dose reduction in affected cases. Table 3 summarizes the data on AEs reported in patients during lorlatinib therapy.

**Table 3:** The spectrum of treatment-related adverse events in patients treated with lorlatinib.

**AEs**	**Overall, *n* (%)**	**Grade 1–2, *n* (%)**	**Grade 3–4, *n* (%)**
Hyperlipidemia	46 (56.1)	43 (52.4)	3 (3.7)
Peripheral edema	17 (20.7)	17 (20.7)	-
Neurocognitive AEs	7 (8.5)	5 (6.1)	2 (2.4)
Cognitive impairment	3 (3.7)	3 (3.7)	-
Mood events	3 (3.7)	2 (2.4)	1 (1.2)
Psychotic events	1 (1.2)	0 (0.0)	1 (1.2)
Weight gain	6 (7.3)	6 (7.3)	-
Peripheral neuropathy	3 (3.7)	3 (3.7)	-
Creatinine increased	2 (2.4)	2 (2.4)	-
AEs leading to dose reduction	14 (17.1)	-	-
AEs leading to dose discontinuation	0 (0.0)	-	-

AEs: adverse events; -: not applicable.

With a median follow-up of 82.7 months, 64 patients had ≥ 12 months of lorlatinib exposure with available AE documentation; among these, no delayed (first occurrence after 12 months) AEs were observed. No previously unreported late grade ≥ 3 toxicities attributable to lorlatinib were identified.

## Discussion

This study on pretreated patients yielded more encouraging results than many previously published datasets (Table 4). The obtained data suggest that differences in the selection of the patients may have a pronounced impact on study outcomes. Our investigation included only those patients who experienced benefit on prior TKI for at least 4 months, developed acquired resistance to this therapy, and eventually received lorlatinib. Other published studies generally did not address the duration of response to prior TKI [11–16]. Several prior datasets have reported that a longer history of benefit on early-generation ALK TKIs is associated with longer PFS on lorlatinib [15, 17–19]. However, this relationship was not observed in some previously published studies [20–22]. Our investigation demonstrates that the major factor determining the success of lorlatinib therapy is the duration of response to previous TKI.

**Table 4:** Lorlatinib in NSCLC patients with prior progression on targeted therapy.

Study	*N*, comments	ECOG PS	Method of ALK testing	Prior ALK TKI treatment	Duration of response to the last TKI	ORR (%)	IC-ORR (%)	PFS (months)	OS (months), defined as the time from lorlatinib initiation to death	OS (months), defined as the time from the diagnosis of metastatic disease to death	Mode recruitment
Present study	82	0/1: 80.5%; 2: 19.5%	PCR/NGS: 43.9% FISH: 45.1% IHC: 11.0%	1 line: 75.6% 2 lines: 19.5% 3 lines: 4.9%	4–12 months: 37.8%; ≥ 12 months: 62.2%	64.6	66.2 (43/65)	All: 66.7 1 prior TKI: 66.7 ≥ 2 prior TKIs: 75.0	All: not reached 1 prior TKI: Not reached ≥ 2 prior TKIs: 65.7	Not reached	Expanded access and routine clinical use
Solomon et al. [7], Ou et al. [11]	198	0/1: 96.4%; 2: 3.6%	FISH or IHC (% not reported)	1 line: 44.0% 2 lines: 32.8% 3 lines: 21.2% ≥ 4 lines: 2.0%	Not reported	47.0	63.0 (51/81)	All: 7.3 Previous crizotinib: not reached Previous non-crizotinib TKI: 5.5 ≥ 2 prior TKIs: 6.9	All: not reached Previous crizotinib: not reached Previous non-crizotinib TKI: 37.4 ≥ 2 prior TKIs: 20.7	Not reported	Clinical trial
Calles et al. [12]	61, with at least one prior 2nd-gen TKI	0/1: 72.1%; 2/3: 13.1%; Unknown: 14.7%	FISH: 54.4%; IHC: 31.1%; PCR/NGS: 5.9%	1 line: 5.3% 2 lines: 36.8% 3 lines: 19.3% ≥ 4 lines: 35.1%	Not reported	32.8	58.8 (10/17)	All: 11.2 1 prior TKI: 15.1 2 prior TKIs: 11.1 ≥ 3 prior TKIs: 7.6	All: 13.5 1 prior TKI: 17.4 2 prior TKIs: 13.5 ≥ 3 prior TKIs: 12.0	45.8	Expanded access
Tian et al. [13]	57	0/1: 87.7%; 2/3: 12.3%	IHC: 35.4% NGS: 40.0% FISH: 1.5% ≥2 methods: 23.1%	1 line: 38.5% ≥ 2 lines: 49.2%	Not reported	49.2	45.2 (19/42)	1 prior TKI: 49.7 ≥ 2 prior TKIs: 12.2	Not reached	Not reported	Not reported
Shih et al. [14]	54, with at least one prior 2nd-gen TKI	0/1: 36.8%; 2: 9.5%; Unknown: 54.0%	Not reported	Patients with at least one 2nd-gen TKI 1 line: 18.5% 2 lines: 50.0% ≥ 3 lines: 31.5%	Not reported	13.7	30.6 (11/36)	All: 9.2 1 prior TKI: not reached 2 prior TKIs: 6.1 ≥ 3 prior TKIs: 8.1	All: not reached 1 prior TKI: not reached 2 prior TKIs: not reached ≥ 3 prior TKIs: 21.2	Not reported	Expanded access
Biswas et al. [15]	38	Not reported	FISH: 50.0%; IHC: 50.0%	Not reported	Not reported	70.3	Not reported	Not reached	Not reported	93.1	Expanded access
Goto et al. [16]	51, with prior alectinib given the 1st line	0/1: 58.9%; 2–4: 17.6%; Unknown: 23.5%	Not reported	1 line of alectinib: 70.6% 1 line of alectinib followed by chemotherapy or another TKI: 29.4%	Not reported	35.7	28.6 (6/21)	11.1	Not reported	Not reported	Not reported
Alexander et al. [17]	38	0/1: 76.0%; ≥ 2: 24.0%; Unknown: 34.2%;	IHC screened, then validated by FISH	1 line of 2nd-gen: 42.1%; ≥ 2 lines of 1st- and 2nd-gen: 42.1%; ≥ 2 lines of 2nd-gen only: 15.8%;	Not reported	44.4	35.3 (6/17)	All: 7.3 1 line of 2nd-gen: 10.5 ≥ 2 lines of 1st- and 2nd-gen: 34.6 ≥ 2 lines of 2nd-gen only: 1.1	All: 19.9 1 line of 2nd-gen: 19.9 ≥ 2 lines of 1st and 2nd-gen: 34.6 ≥ 2 lines of 2nd-gen only: 2.1	All: 45.0 1 line of 2nd-gen: 116.3 ≥ 2 lines of 1st and 2nd-gen: 39.4 ≥ 2 lines of 2nd-gen only: 32.0	Expanded access
Baldacci et al. [18]	208, mainly with prior crizotinib and 2nd-gen TKI	0/1: 60.1%; ≥ 2: 23.1%; Unknown: 16.8%	Not reported	1 line: 9.6% 2 lines: 57.7% ≥ 3 lines: 32.7%	Not reported	49.0	56.3 (90/160)	9.9	32.9	97.3	Expanded access
Hochmair et al. [19]	37	Not reported	IHC: 35.1% FISH: 45.9% NGS: 2.7% ≥ 2 methods: 16.2%	1 line: 27.0% 2 lines: 35.1% 3 lines: 35.1% 4 lines: 2.7%	Not reported	43.2	62.5 (5/8)	Not reported	All: 10.2 1 line of TKI: 6.4 2 lines of TKI: 31.2 ≥ 3 lines of TKI: 7.1	Not reported	Expanded access
Frost et al. [20]	52	0/1: 90.4%; 2: 1.9%; Unknown: 7.7%	Not reported	Not reported	Not reported	42.4	Not reported	7.0	24.7	79.6	Expanded access
Zhu et al. [21]	76	Not reported	FISH: 46.1% IHC: 46.1% NGS: 9.2% PCR: 6.6% ≥ 2 methods in 6 cases	1 line: 13.2%; 2 lines: 59.2%; ≥ 3 lines: 27.6%	Not reported	32.8	34.6 (18/52)	All: 9.3 1 line of TKI: 9.3 2 lines of TKI: not reached ≥ 3 lines of TKI: 6.5	Not reported	All: not reached 5-year OS: 1 line of TKI: 85.7% ≥ 2 line of TKI: 77.9% ≥ 3 line of TKI: 72.5%	Expanded access
Peled et al. [22]	106	0/1: 61.3%; ≥ 2: 14.2%; Unknown: 24.5%	FISH: 76.4%; IHC: 31.1%; NGS: 7.5%; PCR: 13.2%; ≥ 2 methods in some cases	Not reported	Not reported	59.7	61.5 (40/65)	Not reported	Not reported	89.1 months	Expanded access

ALK: anaplastic lymphoma kinase; ECOG PS: Eastern Cooperative Oncology Group Performance Status; FISH: fluorescence in situ hybridization; gen: generation; IC-ORR: intracranial objective response rate; IHC: immunohistochemistry; NGS: next-generation sequencing; NSCLC: non-small cell lung cancer; ORR: objective response rate; OS: overall survival; PCR: polymerase chain reaction; PFS: progression-free survival; TKI: tyrosine kinase inhibitor.

There are multiple factors contributing to ALK TKI resistance. The minority of patients do not respond even to upfront targeted therapy; mistakes in the determination of *ALK* status may have some role in these failures, given that FISH and IHC may sometimes produce erroneous results [23]. The mechanisms of resistance to TKI have been studied with a significant level of comprehension [24]. Some of these pathways are shared by a diverse spectrum of compounds and involve, e.g., accelerated drug efflux or degradation [25]. These features are likely to explain why some patients actually fail to benefit significantly from any administered therapy. Other routes of tumor resistance are specific for a particular class of inhibitors: If the tumor becomes resistant to ALK TKI due to the emergence of *KRAS* or *BRAF* mutation, it will no longer respond to any ALK-targeted drug. The most well-known drug escape pathway is the modification of the target, e.g., the emergence of secondary mutation [26]. This mechanism is drug-specific, i.e., an appropriate change of the drug within a class is likely to render a clinical benefit. Studies on emerging resistance to cetuximab show that class-specific escape mechanisms arise more rapidly than drug-specific ones [27]. Early failure of anti-EGFR therapy is typically driven by *KRAS* mutations, conferring cross-resistance to other anti-EGFR agents [27]. Acquired resistance in long-term responders is often mediated by the *EGFR* S492R mutation, which remains sensitive to panitumumab and therefore supports therapeutic switching [27]. Similarly, the most common cause of acquired resistance to ALK inhibitors in NSCLC is the emergence of secondary *ALK* mutations affecting gatekeeper residues in the kinase domain [28]. Furthermore, each successive generation of ALK TKIs targets an increasing number of these secondary mutations, which allows for prolonged sequential use of various ALK inhibitors [29]. However, no studies to date have systematically examined the relationship between the timing of acquired resistance and drug-specific resistance alterations in ALK-positive NSCLC, highlighting an important area for future research.

The obtained data and the above assumptions call attention to an under-recognized source of bias, which may affect outcomes of the studies of novel drugs. The response duration of at least 4 months to prior TKI was not a formally predefined criterion, but rather emerged due to practical access limitations in Russia. Access to compassionate-use programs involving novel, expensive drugs usually requires significant paperwork with a prolonged turnaround time. The 4-month threshold was chosen post hoc to reflect a clinically meaningful benefit from prior therapy, in line with definitions of acquired resistance in *ALK*-positive NSCLC-progression after 3–6 months of treatment [1]. This requirement preferentially selected patients who were prior responders and thus limits generalizability to populations of primary refractory or rapidly progressing patients.

Lorlatinib is especially effective in overcoming drug-specific mechanisms of acquired resistance that are common with earlier-generation ALK TKIs. It remains effective against tumors harboring crizotinib-, brigatinib-, or alectinib-resistant mutations and demonstrates superior CNS penetration. Consequently, our cohort was enriched for patients whose tumors remained ALK-dependent but failed earlier TKIs due to secondary resistance mutations or CNS progression. The same bias explains why, in our dataset, patients with brain involvement produced better PFS and OS estimates than subjects with visceral-only lesions. Potential contributors to prolonged survival may also include the younger median age of patients in this study (51 years), better ECOG PS (80.5% with 0/1), and the high proportion of patients with brain metastases who received earlier radiotherapy, potentially optimizing CNS control before lorlatinib. These factors may explain why survival outcomes in our study exceed those reported in other real-world series.

The safety profile of lorlatinib in our study was consistent with previous reports [11, 13, 14, 30], showing a high rate of treatment-related AEs, although only 8.1% experienced grade 3 or higher toxicity. The most common AEs reflect the known pharmacologic profile of lorlatinib, which are attributed to its off-target effects on lipid metabolism and neurological activity. It is worth noting that no patients had to permanently discontinue lorlatinib due to toxicity, and less than a quarter of patients required dose reductions, indicating excellent tolerability. Extended follow-up with systematic AE documentation revealed no delayed toxicities occurring after 12 months of therapy, supporting the feasibility of prolonged lorlatinib treatment in responsive patients. This is in contrast to previous ALK inhibitors, where discontinuations due to toxicity were more frequent, and it supports the feasibility of using lorlatinib in routine practice [1, 3].

This study has several limitations. Its retrospective design and enrichment for patients who had previously responded to ALK TKI therapy introduce selection bias, limiting its applicability to broader populations, especially those with primary resistance. The absence of molecular profiling data on resistance mechanisms further restricts mechanistic interpretation. In addition, the single-country setting may limit generalizability to regions with different treatment access pathways and sequencing strategies.

In conclusion, lorlatinib demonstrated marked efficacy in our selected prior-responder cohort; our findings support consideration of lorlatinib for patients who have developed resistance after prolonged benefit to earlier TKIs. These results may not be generalizable to unselected populations. In resource-limited settings with delayed lorlatinib access, prioritizing sequencing for prior responders may optimize outcomes. However, efforts to expedite access are crucial in order to include a broader population and reduce bias.

## Abbreviations

AEs: adverse events
ALK: anaplastic lymphoma kinase
CNS: central nervous system
DCR: disease control rate
ECOG PS: Eastern Cooperative Oncology Group Performance Status
FISH: fluorescence in situ hybridization
HR: hazard ratio
IC-DCR: intracranial disease control rate
IC-ORR: intracranial objective response rate
IHC: immunohistochemistry
NR: not reached
NSCLC: non-small cell lung cancer
ORR: objective response rate
OS: overall survival
PFS: progression-free survival
TKI: tyrosine kinase inhibitor

## Declarations

### Availability of data and materials

The raw data supporting the conclusions of this manuscript will be made available by the corresponding author (a.musaelyan8@gmail.com), without undue reservation, to any qualified researcher.

### Copyright

© The Author(s) 2026.
